# The Telephone in Medical Practice

**Published:** 1915-02-20

**Authors:** 


*To the Editor of* The Hospital.

### The Telephone in Medical Practice.

*To the Editor of* The Hospital.

Sir,—The other day our matron was rung up on the telephone by a medical man, who said that he was about to engage a nurse who had recently left our hospital. He wanted to know if the nurse's character was satisfactory, and he asked a number of other questions concerning her. Our matron immediately told him that she could give him no information on the telephone with regard to a nurse's personal character.

The medical man was very indignant, and said that he would at once "report" our matron's conduct to the board of management. Whereupon our matron told him that he could do just what he liked, and that she hoped he would not trouble her again but address all his inquiries to the board of management. It seems absurd that any medical man should expect a matron to answer confidential inquiries over the telephone.

How was the matron to know that the medical man who was said to be speaking over the telephone was really the person he said he was? Needless to say, no "report" has yet reached the board of management about the matron's incivility. Should it do so, the medical man will sustain a further shock to his dignity.—Yours faithfully, A Hospital Secretary.

